# The rate-pressure product combined model within 24 h on admission predicts the 30-day mortality rate in conservatively treated patients with intracerebral hemorrhage

**DOI:** 10.3389/fneur.2024.1377843

**Published:** 2024-06-07

**Authors:** Hui Zheng, Yuguang Tang, Hai Zhou, Xiang Ji

**Affiliations:** Department of Neurosurgery, The Second Affiliated Hospital, Chongqing Medical University, Chongqing, China

**Keywords:** intracerebral hemorrhage, rate-pressure product, ICH score, prognostic model, GCS-Glasgow Coma Scale

## Abstract

**Background and objectives:**

Recently, some literature has proposed new indicators such as rate-pressure product, platelet-to-lymphocyte ratio, neutrophil-to-lymphocyte ratio, etc. However, there has been no literature that has utilized these new indicators to establish a predictive model for assessing the risk of mortality in patients within 24 h on admission. Therefore, this study aims to build a predictive model that can rapidly assess the likelihood of mortality in patients within 24 h of admission.

**Methods:**

The datasets used in this study are available from the corresponding author upon reasonable request. Patients were randomly assigned to the training or validation cohort based on a ratio of 7:3, which was implemented as internal validations for the final predictive models. In the training set, least absolute shrinkage and selection operator (LASSO) regression was employed to select predictive factors, followed by both univariate and subsequent multivariate analysis. The predictive ability was assessed by the area under the receiver operating characteristic (ROC) curve.

**Results:**

A total of 428 patients were included in our research. The final model included 4 independent predictors (Glasgow Coma Scale, hematoma volume, rate-pressure product, c-reactive protein) and was developed as a simple-to-use nomogram. The training set and internal validation set model’s C-index are 0.933 and 0.954, demonstrating moderate predictive ability with regard to risks of mortality. Compared to ICH score (AUC: 0.910 and 0.925), the net reclassification index (NRI) is 0.298 (CI = −0.105 to 0.701, *p*: 0.147) and integrated discrimination improvement (IDI) is 0.089 (CI = −0.049 to 0.228, *p*: 0.209). Our model is equally excellent as the classic ICH score model.

**Conclusion:**

We developed a model with four independent risk factors to predict the mortality of ICH patients. Our predictive model is effective in assessing the risk of mortality in patients within 24 h on admission, which might be worth considering in clinical settings after further external validation.

## Introduction

Intracerebral hemorrhage (ICH) is a type of stroke caused by bleeding within the brain tissue (1). It is a severe and potentially life-threatening condition that can lead to significant neurological impairment or even death. The case fatality rate of intracerebral hemorrhage (ICH) is high (40% at 1 month and 54% at 1 year), and only 12 to 39% of survivors achieve long-term functional independence (2). Therefore, it is necessary to improve the ability to ICH patients during early period and provide timely interventions to improve patient outcomes. Currently, numerous predictive models have been applied to predict the outcome of death in intracerebral hemorrhage. However, there are few models capable of rapid prediction within 24 h of admission.

Rate-pressure product (RPP) as a classical markers is used to measure the load of the heart through the product of heart rate and systolic blood pressure (3). RPP has demonstrated significant predictive capability for disease progression and prognosis in both cardiovascular disease and aSAH. Previous studies have suggested that higher heart rate and blood pressure levels indicate autonomic dysfunction, independently associated with poorer one-year prognosis and reduced survival in patients with intracerebral hemorrhage (ICH) (4–9). However, its role in intracerebral hemorrhage remains unclear.

Therefore, we have incorporated new indicators and constructed a relevant predictive model to predict the 30-day mortality. Our predictive model shows good predictive performance in ICH during early stage.

## Materials and methods

### Patients

This retrospective study was approved by the local institutional review board (The National Drug Clinical Trial Institution). The data for this study was from inpatients admitted to the neurosurgery and critical care departments at Chongqing Medical University Affiliated Hospital from 1 January 2015 to 31 December 2022.

The inclusion criteria for our cohort were as follows:

1. Individuals with acute intracerebral hemorrhage (ICH) diagnosed by the presence of a sudden focal neurological deficit, with visible brain hematoma in a head computed tomography (CT) scan conducted within 24 h of admission.
2. Availability of initial blood pressure and heart rate measurements recorded within the first 24 h from the onset of symptoms.
3. Age >18 years old.

In consideration of the significant impact of surgery on the mortality rate of critically ill patients, we excluded surgical patients and focused solely on those receiving conservative treatment for our study. Additional exclusion criteria included:

1. Absence of spontaneous breathing.
2. More than 24 h elapsed from symptom onset to hospital admission.
3. Patients without complete inpatient information records.
4. Hematomas caused by head trauma, arteriovenous malformations, aneurysms, cerebral vein and sinus thrombosis, neoplasms, hemorrhagic diathesis, anticoagulant therapy, or illicit drug abuse.
5. The patient underwent surgical treatment after admission.

### Clinical management

All patients with suspected intracerebral hemorrhage first underwent radiographic imaging to identify the source of bleeding. Relevant vital signs and hematological indicators are recorded within 24 h. All patients with ICH received antifibrinolytic drugs for one time and the systolic blood pressure (SBP) of patients with intracerebral hemorrhage (ICH) is lowered to 160 mmHg early and steadily lowered to a target range of 130–140 mmHg to avoid excessive fluctuations. Critical patients were placed in the ICU for further treatment.

### Data collection

Demographic data, including age and gender, Glasgow Coma Scale (GCS) scores, vital signs (temperature, systolic and diastolic blood pressure, respiration rate, and heart rate), medical history (such as diabetes mellitus, history of ischemic or hemorrhagic stroke, atrial fibrillation, and prior use of antiplatelet medications), risk factors for intracerebral hemorrhage (including hypertension and smoking status), and the time from symptom onset to admission were collected. Additionally, CT findings, such as hematoma location (infratentorial or supratentorial), presence of intraventricular hemorrhage, and volume of intracerebral hemorrhage (calculated using the ABC/2 method from head CT scans) were recorded. Laboratory results for myoglobin, troponin, white blood cell count (WBC), serum sodium, and serum potassium were obtained, with only initial laboratory findings and neuroimaging results after admission being used in the analysis. The initial recordings of all vital signs and laboratory indicators were included in our study. Information regarding whether surgical treatment was performed after intracerebral hemorrhage was also documented. The ICH score is calculated based on the volume of the hemorrhage, GCS score, hematoma location, and age. In our study, admission rate-pressure product (RPP) was calculated by multiplying the admission systolic blood pressure by the admission heart rate. If there is missing data in the patient records, such as loss to follow-up for outcomes or missing laboratory indicators, we exclude the patient from the analysis.

### Candidate predictors

Based on the current published literature, we included as comprehensive a set of variables as possible. All part of candidate predictors were collected on the day of admission, which included the demographic data (age, gender), hemorrhage features (locations, sizes), medical history (hypertension, diabetes, chronic bronchitis, history of stroke), lifestyle (smoke and drink), blood pressure (SBP and DBP) on admission. The Physical examinations (GCS score), neuroimage evaluated by CT or CTA (volume of hemorrhage, location of hematoma), Measurement of hemorrhage volume assessed according to CT were all written up. The blood pressure (SBP and DBP) was collected from care records in ICU or general wards. All candidate predictors were assessed before the outcome assessment.

### Outcome assessment

Our primary clinical outcome was mortality at 30 days. Relevant death information was obtained from electronic medical records. Follow-up telephone calls to obtain relevant information if the patient is discharged early.

### Sample size

Because this study was descriptive in nature, power calculations were not performed, and selection criteria were used to establish the final cohort size. Sample size adequacy was assessed based on confidence intervals for the primary endpoint estimates.

### Statistical analysis

The dataset obtained from the first center was randomly divided into training and validation cohorts at a 7:3 ratio as training set and validation set and the variables were compared. Non-normally distributed data was presented as median (interquartile range). Categorical variables were analyzed using the chi-square test or Fisher’s exact test, while continuous variables were examined using student’s *t*-test or rank-sum test in univariate analysis. Diagnostic analyses of the final model included an examination of nonlinear relationships (evaluated by RCS regression), influential points (assessed by Cook’s distance), and multicollinearity (detected by the variance inflation factor of each covariate). In the training cohort, multivariate analysis was carried out using the least absolute shrinkage and selection operator (LASSO) logistic regression to screen independent risk factors and construct a predictive nomogram for ICH. The performance of the nomogram was assessed using the receiver operating characteristic (ROC) curve and calibration curve, with the area under the ROC curve (AUC) ranging from 0.5 (no discriminant ability) to 1 (complete discriminant ability). Results with a *p*-value of <0.05 were considered statistically significant. IDI, NRI and likelihood ratio test was used to compare new model with ICH score.

All statistical analyses were conducted using R software (version 4.2.2). This study adhered to the TRIPOD (transparent reporting of a multivariable prediction model for the individual prognosis or diagnosis) statement for reporting.

## Results

### Study population

**Figure 1:**
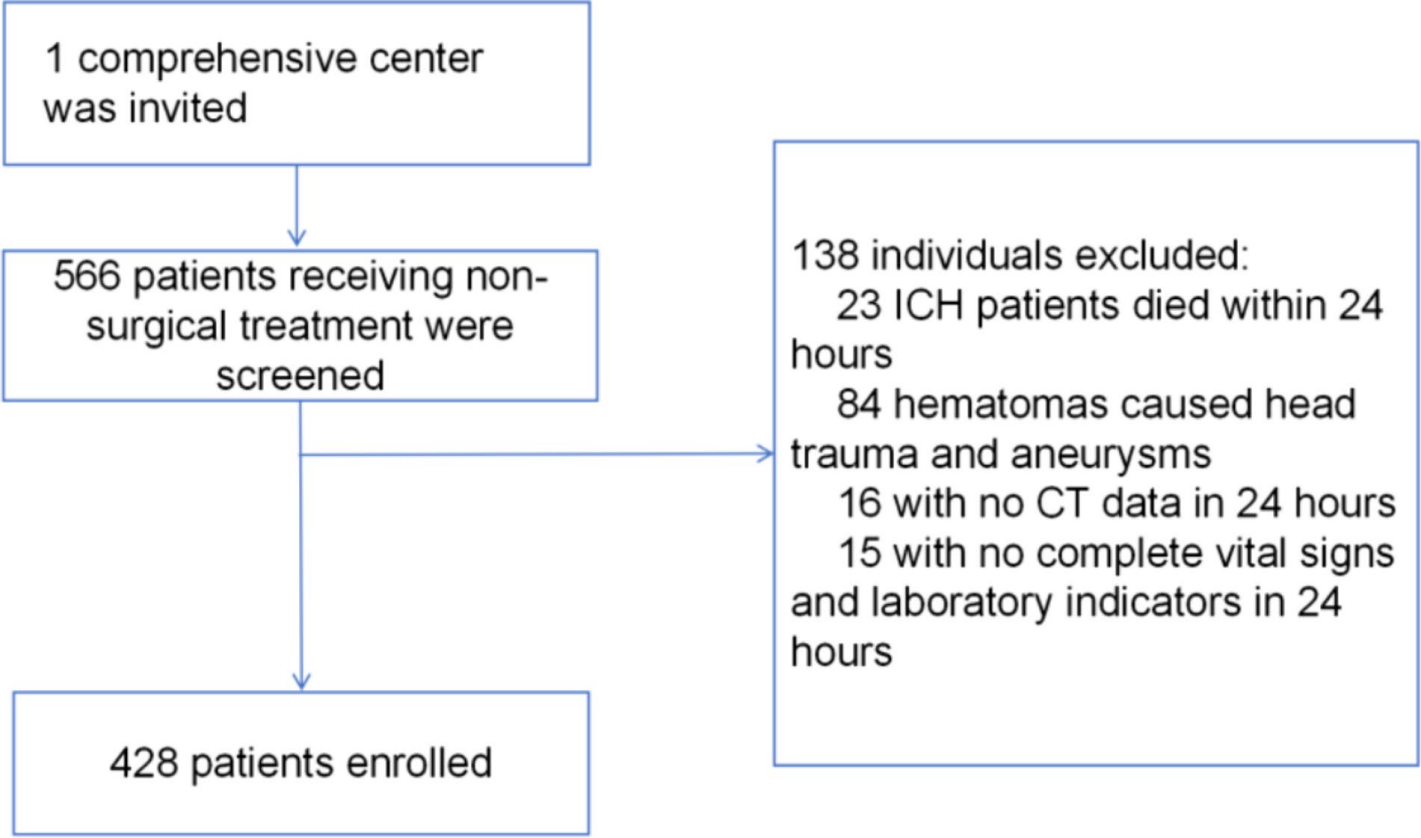
The procession of the cohort assemble.

The baseline demographic and clinical characteristics of the study population are presented in Table 1. In this study, we analyzed the baseline demographic and clinical characteristics of the cohorts, a training cohort consisting of 300 individuals and an internal test cohort comprising 128 individuals. Of these, 82 (19%) patients died within 30 days after intracerebral hemorrhage. In Table 1, we compared training cohort with test cohort. These findings suggested that the baseline demographic and clinical characteristics were generally well-balanced between the training and internal test cohorts, thus supporting the comparability and generalizability of the predictive model developed in this study. An overview of the development and validation cohort assembly process is shown in Figure 1.

**Table 1:** Patient demographics and baseline characteristics.

Characteristic	Cohort		*p*-value
	Training cohort, *N* = 300	Internal test cohort, *N* = 128	
Age (years)			0.662
Median (IQR)	67 (56, 77)	66 (56, 75)	
Gender			0.734
Male	192 (64.0%)	52 (40.6%)	
Female	108 (36.0%)	76 (59.3%)	
Hematoma location			0.492
Supratentorial origin	262 (87.3%)	116 (90.6%)	
Infratentorial origin	38 (12.7%)	12 (9.4%)	
IVH			0.318
No	114 (38.0%)	58 (45.3%)	
Yes	186 (62.0%)	70 (54.7%)	
Smoking			0.577
No	180 (60.0%)	82 (64.1%)	
Yes	120 (40.0%)	46 (35.9%)	
Drinking			0.876
No	228 (76.0%)	98 (75.0%)	
Yes	72 (24.0%)	32 (25.0%)	
Diabetes			0.485
No	272 (90.7%)	112 (87.5%)	
Yes	28 (9.3%)	6 (12.5%)	
SBP (mmHg)			0.143
Mean ± SD	164 ± 32	171 ± 28	
DBP (mmHg)			0.201
Mean ± SD	95 ± 18	99 ± 18	
RPP			0.895
Median (IQR)	12,872 (10,868, 16,004)	12,935 (11,169, 14,855)	
GCS			0.065
Median (IQR)	13.0 (8.0, 15.0)	14.0 (11.0, 15.0)	
Hematoma volume (mL)			0.055
Median (IQR)	18 (6, 45)	11 (5, 24)	
K (mmol/L)			0.382
Mean ± SD	3.67 ± 0.46	3.74 ± 0.51	
Na (mmol/L)			0.447
Median (IQR)	138.1 (135.7, 140.7)	137.6 (135.6, 140.2)	
Albumin (g/L)			0.578
Median (IQR)	41.8 (39.5, 45.7)	42.1 (39.2, 43.9)	
Hb (g/L)			0.517
Median (IQR)	136 (124, 146)	136 (125, 143)	
WBC (K/μL)			0.070
Median (IQR)	9.5 (7.3, 12.9)	8.4 (6.6, 11.1)	
NEU (K/μL)			0.061
Median (IQR)	8.4 (5.5, 11.4)	6.5 (5.4, 9.3)	
LYM (K/μL)			0.667
Median (IQR)	0.99 (0.66, 1.32)	0.92 (0.59, 1.27)	
MON (K/μL)			0.391
Median (IQR)	0.42 (0.31, 0.60)	0.38 (0.29, 0.57)	
PLT (K/μL)			0.631
Median (IQR)	184 (153, 231)	172 (153, 240)	
CRP (mg/L)			0.165
Median (IQR)	5 (5, 12)	5 (5, 7)	
SIRI			0.216
Median (IQR)	3.6 (1.8, 7.3)	2.7 (1.5, 5.6)	
PLR			0.967
Median (IQR)	197 (144, 292)	200 (130, 308)	
NLR			0.371
Median (IQR)	9 (5, 14)	9 (4, 14)	
INR			0.325
Median (IQR)	0.98 (0.93, 1.06)	0.99 (0.95, 1.06)	

^1^*n* (%).^2^Wilcoxon rank sum test; Pearson’s chi-squared test; Welch two sample *t*-test.

### Predictive model

**Figure 2:**
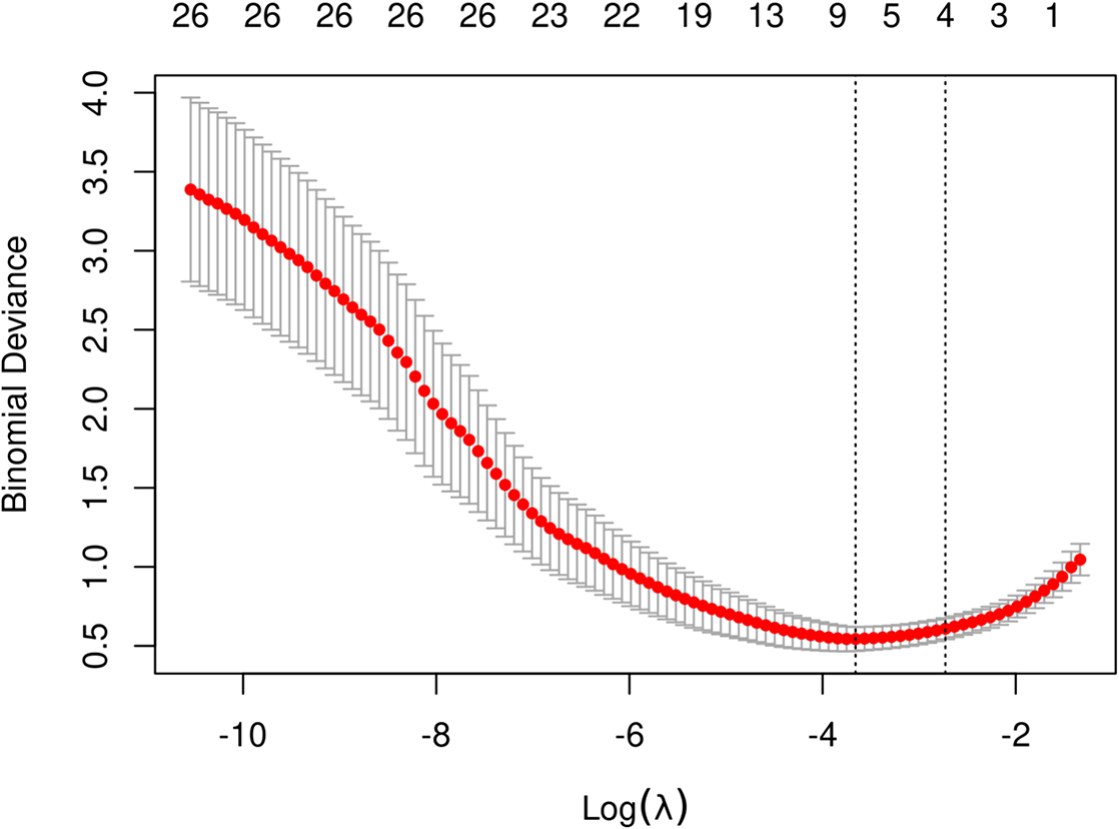
LASSO regression cross-validation plot.

**Figure 3:**
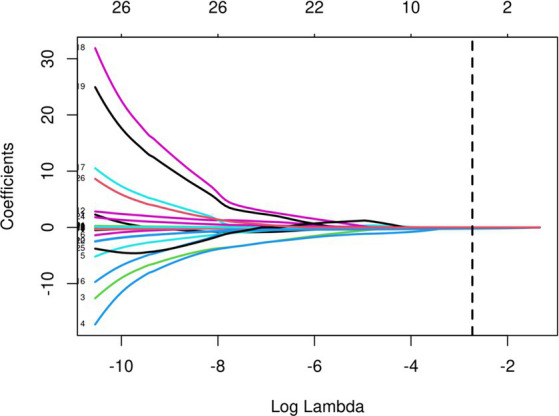
LASSO regression coefficient path plot.

**Figure 4:**
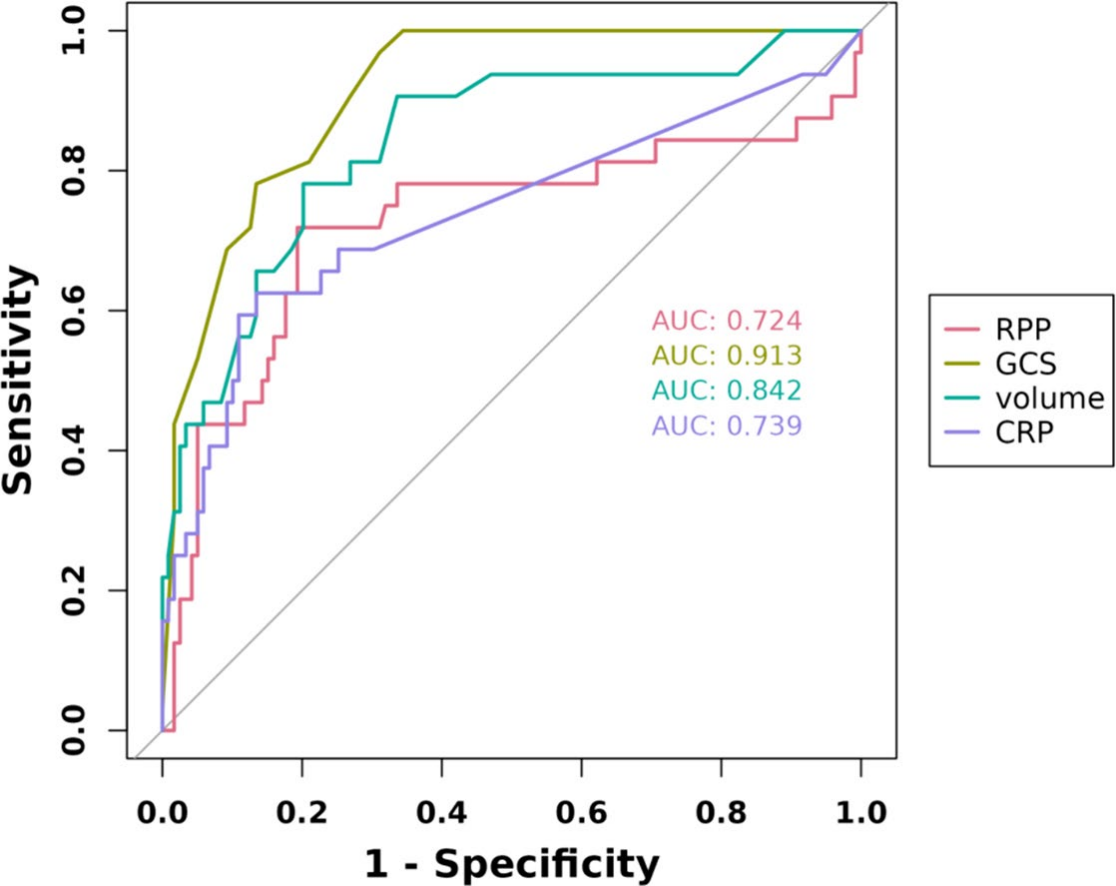
The receiver operating characteristic curves of 4 candidate diagnostic indicators.

**Figure 5:**
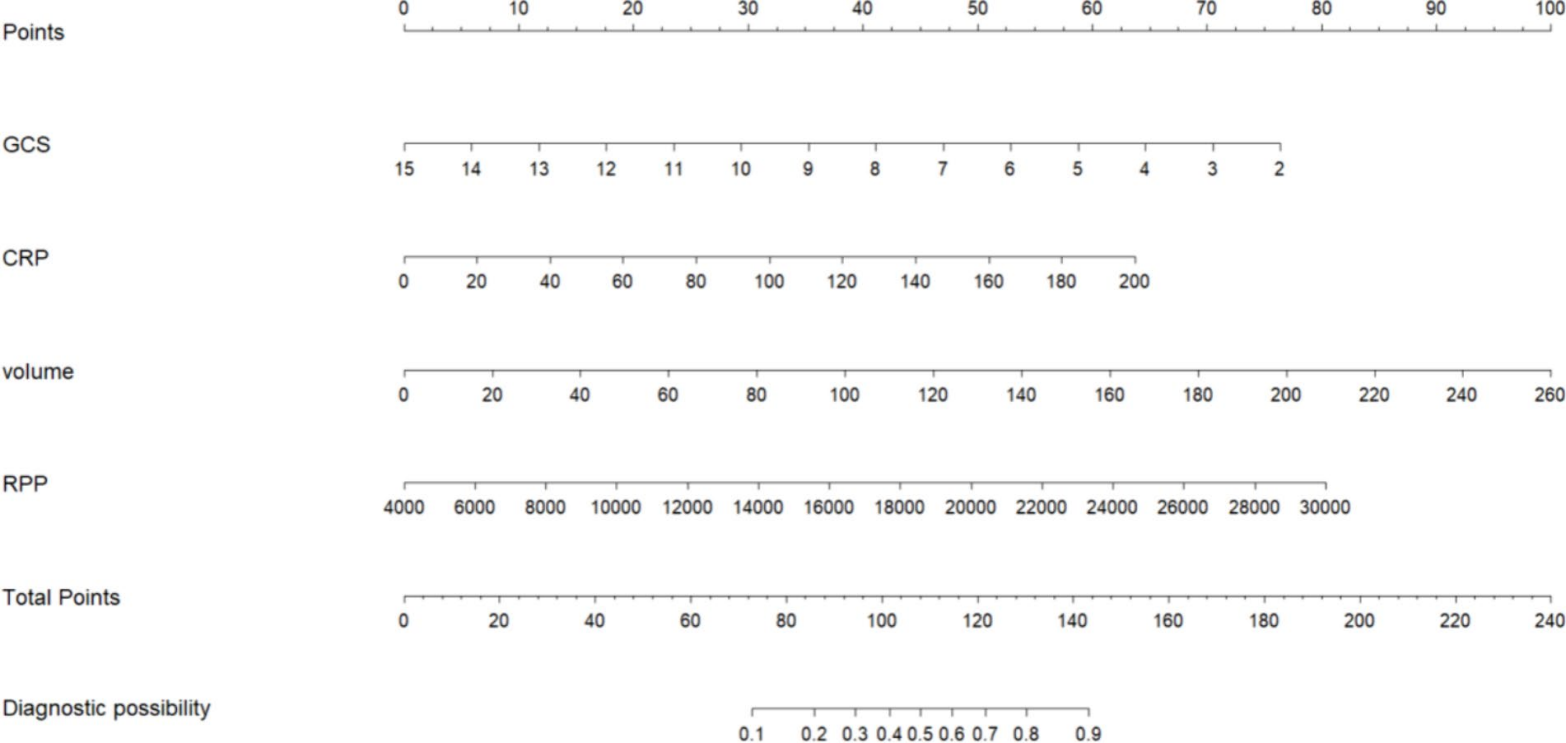
Nomogram of prediction model.

The initial model incorporated all candidate predictors. Through LASSO regression analysis in the training cohort, this set was reduced to 4 potential predictors. The coefficients are detailed in Supplementary Table S1, and a coefficient profile is presented in Figure 2. Additionally, a cross-validated error plot of the LASSO regression model is depicted in Figure 3. The coefficients for each predictor and intercept in the multivariate logistic model are presented in Table 2. The most regularized and parsimonious model, with a cross-validated error within one standard error of the minimum, included 4 variables. As demonstrated in Figure 4, ROC analysis of the aforementioned variables resulted in AUC values exceeding 0.5. Univariate analyses were utilized to compare the indices between different outcome groups. Subsequent multivariate logistic analyses were performed in different cohorts. The final logistic model comprised 4 independent predictors (RPP, CRP, GCS, hemorrhage volume). The risk of death is 1.02 times higher in individuals with higher RPP (OR: 1.02, 95% CI: 1.01–1.04). A higher GCS score is a protective factor (OR: 0.68, 95% CI: 0.54–0.82). The risk of death is lower by 1.03 times in individuals with higher hematoma volume (OR: 1.03, 95% CI: 1.01–1.05). Higher CRP levels are associated with a higher death risk by 1.05 times (OR: 1.05, 95% CI: 1.01–1.10). Model was developed as a user-friendly nomogram, presented in Figure 5.

**Table 2:** Results of multivariate logistic regression for training cohort.

Characteristic	*N*	Event *N*	OR1	95% CI1	*p*-value
RPP	300	64	1.02	1.01, 1.04	0.002
GCS	300	64	0.68	0.54, 0.82	<0.001
volume	300	64	1.03	1.01, 1.05	0.009
CRP	300	64	1.05	1.01, 1.10	0.015

### Development and validation of the prediction nomogram

**Figure 6:**
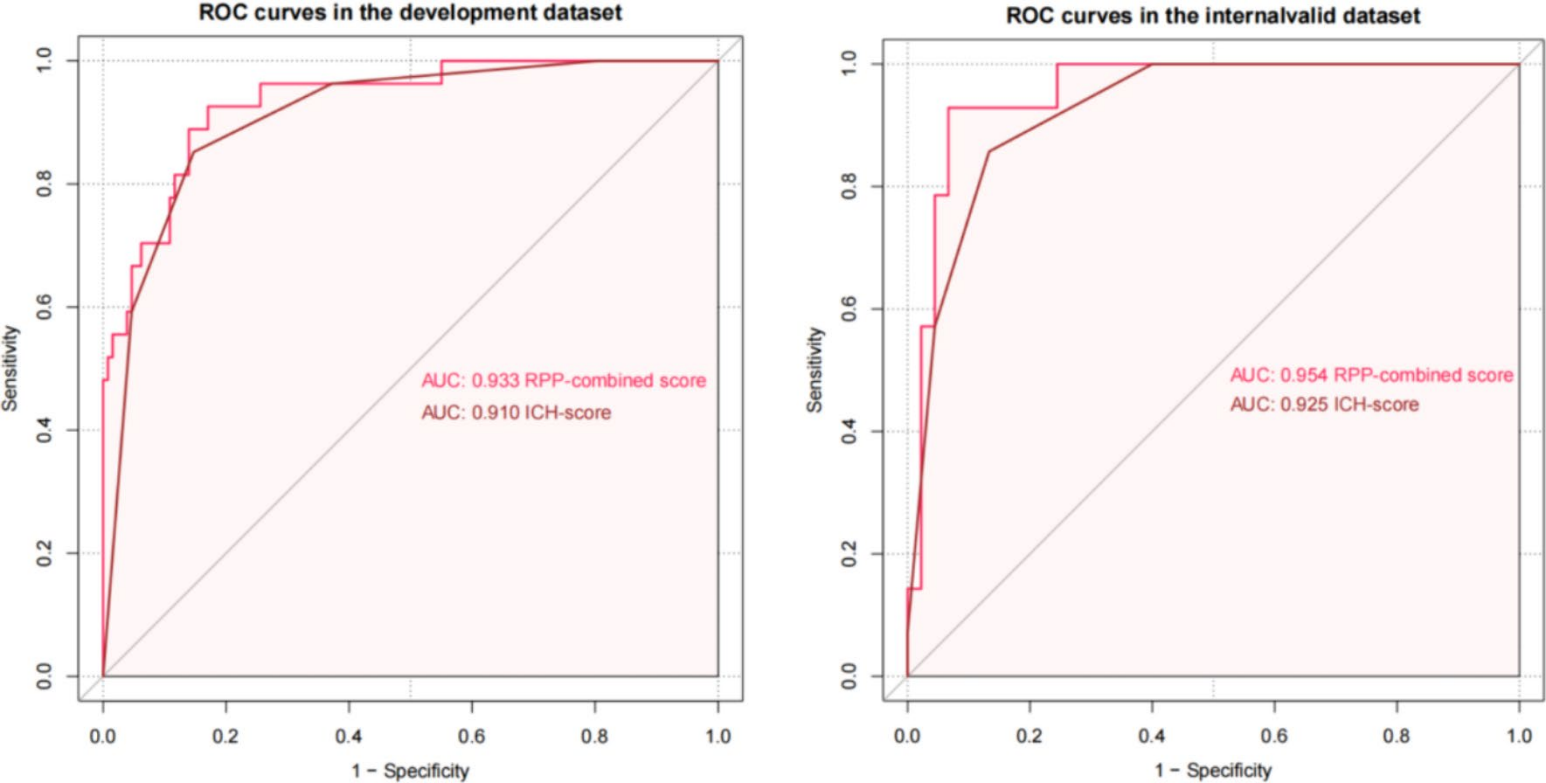
The receiver operating characteristic curve for the developed model and ICH score.

**Figure 7:**
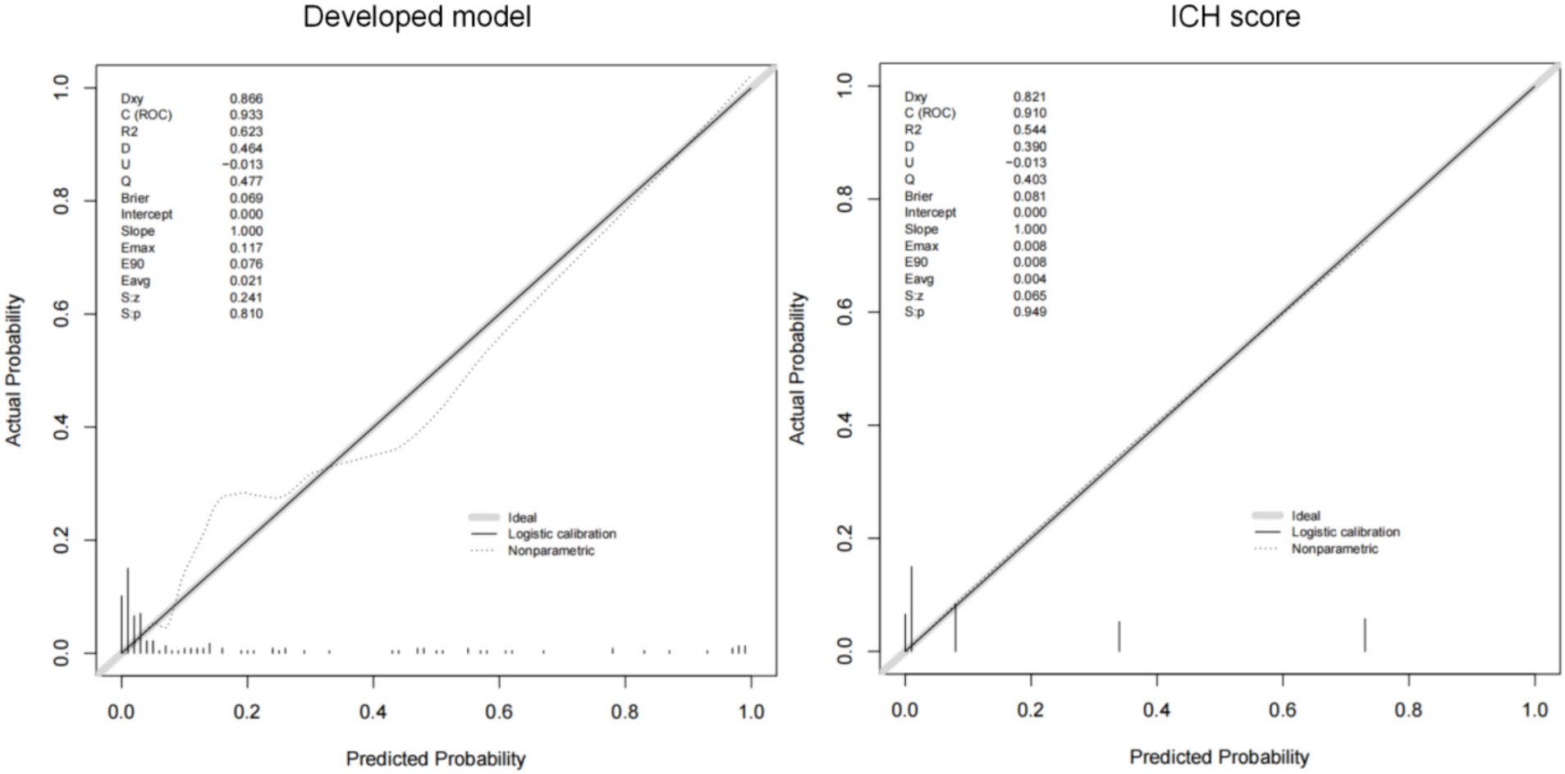
The calibration curve for the developed model and ICH score.

The AUCs of the developed model and ICH score in the different cohorts are illustrated in Figure 6. Calibration plots of the nomogram in the different models are presented in Figure 7, showing a strong correlation between the observed and predicted mortality. The findings indicate that the original nomogram remained suitable for use, with the calibration curve of the model closely approximating the ideal curve, suggesting consistency between the predicted results and actual observations.

### Compared to ICH score

Comparisons between the newly developed scoring model and ICH score were performed. The newly developed model presented a insignificantly improved AUC (AUC = 0.954, 95% CI = 0.90–1) compared to the ICH score (AUC = 0.925, 95%CI = 0.861–0.989, *p*-value of DeLong test = 0.234), in the development cohort. Compared to ICH score, the NRI is 0.298 (CI = −0.105 to 0.701, *p*: 0.147) and IDI is 0.089 (CI = −0.049 to 0.228, *p*: 0.209). The likelihood ratio test for two models was used to assessed. The test statistic is 11.467 with 3 degrees of freedom and a *p*-value of 0.009449516. Our model is equally excellent as the classic ICH score model. The classification accuracy for prediction at different risk cutoff points for the developed model and other metrics such as sensitivity, specificity, positive predictive value (PPV), negative predictive value (NPV), accuracy, precision, recall, and F1 score have been used for model evaluation which are presented in Supplementary Tables S2, S3. Our developed model and ICH score demonstrated good predictive performance.

## Discussion

Intracerebral hemorrhage (ICH) is a critical medical condition associated with high morbidity and mortality rates. Early recognition of patients at high risk of clinical deterioration is of paramount importance in improving outcomes and optimizing treatment strategies. In the past, the ICH score has been widely used as an effective predictive tool; however, it primarily focuses on reflecting brain damage and does not consider the overall systemic status. In recent years, there have been more new biomarkers proposed in stroke, such as CRP, NLR, and RPP, which represent inflammatory and cardiac load markers. We aim to utilize these biomarkers to construct a predictive model that reflects the systemic status of patients with intracerebral hemorrhage upon admission, allowing for the rapid prediction of mortality risk. So we systematically analyzed various indicators upon patient admission and subsequently developed a model with strong predictive performance.

The rate-pressure product (RPP) is a physiological index utilized to assess cardiac workload, calculated as the product of heart rate and systolic blood pressure. Widely employed in clinical and exercise physiology contexts, RPP serves as a valuable tool for evaluating cardiac load and predicting the risk of cardiovascular events (10, 11). Elevated RPP values are often indicative of increased cardiac load and heightened susceptibility to cardiovascular disease. One literature supposed that RPP on admission to be independently associated with in-hospital mortality after aSAH (12). Individuals with severe traumatic brain injury demonstrate diverse myocardial workload patterns that are closely associated with mortality (13). The interaction between the brain and the heart is currently a research hotspot in the field of stroke. After intracerebral hemorrhage, the disruption of the blood-brain barrier leads to the activation of the hypothalamic-pituitary-adrenal axis and the sympathetic nervous system, triggering a systemic inflammatory response (14). The release of inflammatory mediators leads to vasoconstriction in peripheral blood vessels and an increase in heart rate, resulting in elevated rate-pressure product (RPP) index, undoubtedly significantly increasing the burden on the heart. A study supposed that stroke (ischemic stroke, intracerebral hemorrhage, and subarachnoid hemorrhage) can induce neurovascular uncoupling and disrupt cerebral autoregulation, resulting in direct dependence of cerebral blood flow on cardiac function (15). In general, high RPP means high systolic blood pressure and high heart rate. This may imply elevated intracranial pressure and cerebral hypoperfusion following intracerebral hemorrhage, leading to dysregulation of cerebral autoregulation. At this point, cerebral perfusion relies solely on compensatory mechanisms from cardiac function. Therefore, early rate-pressure product (RPP) also serve as a significant indicator reflecting cerebral perfusion. In our study, an elevated rate-pressure product (RPP) upon admission is an independent risk factor for 30-day mortality. The rate-pressure product (RPP), as one of the predictive factors in the predict model, has demonstrated strong predictive capability.

The Glasgow Coma Scale (GCS) and hematoma volume are classical predictive factors for forecasting the prognosis of intracerebral hemorrhage patients (16–19). At present, ICH score is a simple and useful tool to predict the mortality of cerebral hemorrhage (20). By combining the Glasgow Coma Scale score, age, infratentorial origin of ICH, and ICH volume to score patients, the aim is to predict patient prognosis. In recent years, there has been significant development in ICH scoring systems. The max-ICH score, compared to the ICH score, demonstrates more robust predictive capability, with its effectiveness validated in international cohorts (21). Similar to hematoma volume, imaging biomarkers have been widely studied in diseases such as stroke. For example, quantitative susceptibility mapping has an excellent predictive role in stroke (22, 23). Our model is comparable to the ICH score, and even exhibits a better fit. More importantly, our predictive model incorporates vital signs upon admission, enabling early assessment of mortality probability in patients and providing timely guidance for subsequent treatment. As our study aimed to identify early critical patients and avoid surgical interference with outcomes, we included only patients undergoing conservative treatment. Some researchers supposed that Surgical timing between 12 and 26 h after ICH was associated with favorable outcomes (24). Therefore, our scoring system can aid in the early identification of critical patients and serve as a reference for the appropriate timing of surgical intervention for patients.

C-reactive protein (CRP) has been widely studied as an inflammatory marker in intracerebral hemorrhage (25–27). Individual elevation of CRP was associated with poor outcomes. Studies have shown that inflammatory markers upon admission can predict early mortality in patients (28). Preclinical and clinical trials have provided insights into the etiology of intracerebral hemorrhage (ICH) and the mechanisms of injury, highlighting the intricate interplay between edema, inflammation, iron-induced injury, and oxidative stress (29). In our study, we observed a correlation between elevated CRP levels, poorer Glasgow Coma Scale (GCS) scores, and higher mortality rates in patients, which aligns with previous research findings. As one of the indicators in our proposed predictive model, CRP has demonstrated strong predictive capability.

Based on our research, we built a prediction model for early mortality in ICH. In patients after ICH with a higher mortality, our predictive model showed better predictive performance. A nomogram is used to visualize our findings. Our developed predictive model comprehensively evaluates the general condition of patients based on vital signs, systemic inflammatory status, imaging characteristics, and consciousness status. By combining vital signs upon admission with imaging features, we can identify critical patients early and consider intervention. Clinicians can identify high-risk patients early based on our research and provide therapeutic intervention to these patients to improve the patient’s prognosis.

The retrospective nature of this study presents a significant limitation as it may have resulted in the misclassification of mortality and failure to identify key comorbidities. Moreover, our study sample size was not large enough. RCTs, multicenter study and more advanced statistical methods may need to prove clinically useful to individualize mortality in ICH patients.

## Conclusion

We developed a model with four independent risk factors to predict the mortality in ICH. These factors include RPP, CRP, GCS, hemorrhage volume. We explored the significance of RPP in ICH patients. Elevated RPP on admission may indicate that high risk of mortality in ICH. Our constructed predictive model is equally excellent as the classic ICH score.

## Data availability statement

The original contributions presented in the study are included in the article/Supplementary material, further inquiries can be directed to the corresponding author.

## Ethics statement

The studies involving humans were approved by the National Drug Clinical Trial Institution of the Second Affiliated Hospital of Chongqing Medical University. The studies were conducted in accordance with the local legislation and institutional requirements. The human samples used in this study were acquired from a by-product of routine care or industry. Written informed consent for participation was not required from the participants or the participants’ legal guardians/next of kin in accordance with the national legislation and institutional requirements.

## Author contributions

HuZ: Investigation, Methodology, Writing – original draft, Writing – review & editing. YT: Conceptualization, Data curation, Formal analysis, Writing – review & editing. HaZ: Conceptualization, Data curation, Writing – review & editing. XJ: Conceptualization, Data curation, Supervision, Validation, Writing – review & editing.

## Glossary

aSAH: Aneurysmal subarachnoid hemorrhage
DM: Diabetes mellitus
NEU: Neutrophil
LYM: Lymphocyte
MON: Monocyte
PLT: Platelet
SIRI: Systemic immune-inflammation index
PLR: Platelet-to-lymphocyte ratio
NLR: Neutrophil-to-lymphocyte ratio
INR: International normalized ratio
ICH: Intracerebral hemorrhage
AUC: Area under the curve
ROC: The receiver operating characteristic
CRP: c-reactive protein
CT: Computed tomography
DBP: Diastolic blood pressure
GCS: Glasgow Coma Scale
HR: Heart rate
IQRs: Interquartile ranges
ORs: Odds ratios
RPP: Rate-pressure product
SBP: Systolic blood pressure
SD: Standard deviation
WBC: White blood cell
IVH: Intraventricular hemorrhage
