# Supplementary material for: The rate-pressure product combined model within 24 h on admission predicts the 30-day mortality rate in conservatively treated patients with intracerebral hemorrhage
Source: Front Neurol. 2024 Jun 7;15:1377843. doi: 10.3389/fneur.2024.1377843 (PMC11190339; doi:10.3389/fneur.2024.1377843)
Supplement: Supplementary file 1 [file Table_1.DOCX]

Table :The coefficients of Lasso regression analysis

| Coefficient | variable |
| --- | --- |
| 0.0529103266 | (Intercept) |
| 0.0000000000 | gender_level_1 |
| 0.0000000000 | age_level_ |
| 0.0000000000 | Hypertension_level_0 |
| 0.0000000000 | Diabetes_level_0 |
| 0.0000000000 | drinking_level_0 |
| 0.0000000000 | HR_level_ |
| 0.0000000000 | SBP_level_ |
| 0.0000000000 | DBP_level_ |
| 0.0000315103 | RPP_level_ |
| -0.2338596830 | GCS_level_ |
| 0.0087712437 | volume_level_ |
| 0.0000000000 | K_level_ |
| 0.0000000000 | Na_level_ |
| 0.0000000000 | Albumin_level_ |
| 0.0000000000 | Hb_level_ |
| 0.0000000000 | WBC_level_ |
| 0.0000000000 | NEU_level_ |
| 0.0000000000 | LYM_level_ |
| 0.0000000000 | MON_level_ |
| 0.0000000000 | PLT_level_ |
| 0.0056995347 | CRP_level_ |
| 0.0000000000 | SIRI_level_ |
| 0.0000000000 | PLR_level_ |
| 0.0000000000 | NLR_level_ |
| 0.0000000000 | INR_level_ |
| 0.0000000000 | location_level_0 |
| 0.0000000000 | IVH_level_1 |

Table 2 :Classification accuracy for prediction at different risk cutoff points for the developed model

| Risk score threshold | Linear Predictor Cutoff Point | Sensitivity (%) | Specificity (%) | PPV (%) | NPV (%) | Accuracy (%) | Precision (%) | Recall (%) | F1 |
| --- | --- | --- | --- | --- | --- | --- | --- | --- | --- |
| ≥ 0% | -Inf | 100.0 | 0.0 | 21.2 |  | 21.2 | 21.2 | 100.0 | 0.350 |
| ≥ 10% | -2.1972246 | 93.8 | 79.8 | 55.6 | 97.9 | 82.8 | 55.6 | 93.8 | 0.698 |
| ≥ 20% | -1.3862944 | 87.5 | 85.7 | 62.2 | 96.2 | 86.1 | 62.2 | 87.5 | 0.727 |
| ≥ 30% | -0.8472979 | 81.2 | 90.8 | 70.3 | 94.7 | 88.7 | 70.3 | 81.2 | 0.754 |
| ≥ 40% | -0.4054651 | 78.1 | 90.8 | 69.4 | 93.9 | 88.1 | 69.4 | 78.1 | 0.735 |
| ≥ 50% | 0.0000000 | 71.9 | 94.1 | 76.7 | 92.6 | 89.4 | 76.7 | 71.9 | 0.742 |
| ≥ 60% | 0.4054651 | 59.4 | 97.5 | 86.4 | 89.9 | 89.4 | 86.4 | 59.4 | 0.704 |
| ≥ 70% | 0.8472979 | 59.4 | 100.0 | 100.0 | 90.2 | 91.4 | 100.0 | 59.4 | 0.745 |
| ≥ 80% | 1.3862944 | 50.0 | 100.0 | 100.0 | 88.1 | 89.4 | 100.0 | 50.0 | 0.667 |
| ≥ 90% | 2.1972246 | 40.6 | 100.0 | 100.0 | 86.2 | 87.4 | 100.0 | 40.6 | 0.578 |
| ≥ 100% | Inf | 0.0 | 100.0 |  | 78.8 | 78.8 |  | 0.0 |  |

Table 3:Classification accuracy for prediction at different risk cutoff points for the ICH score

| Risk score threshold | Linear Predictor Cutoff Point | Sensitivity (%) | Specificity (%) | PPV (%) | NPV (%) | Accuracy (%) | Precision (%) | Recall (%) | F1 |
| --- | --- | --- | --- | --- | --- | --- | --- | --- | --- |
| ≥ 0% | -Inf | 100.0 | 0.0 | 21.2 |  | 21.2 | 21.2 | 100.0 | 0.350 |
| ≥ 10% | -2.1972246 | 87.5 | 82.4 | 57.1 | 96.1 | 83.4 | 57.1 | 87.5 | 0.691 |
| ≥ 20% | -1.3862944 | 87.5 | 82.4 | 57.1 | 96.1 | 83.4 | 57.1 | 87.5 | 0.691 |
| ≥ 30% | -0.8472979 | 87.5 | 82.4 | 57.1 | 96.1 | 83.4 | 57.1 | 87.5 | 0.691 |
| ≥ 40% | -0.4054651 | 71.9 | 93.3 | 74.2 | 92.5 | 88.7 | 74.2 | 71.9 | 0.730 |
| ≥ 50% | 0.0000000 | 71.9 | 93.3 | 74.2 | 92.5 | 88.7 | 74.2 | 71.9 | 0.730 |
| ≥ 60% | 0.4054651 | 71.9 | 93.3 | 74.2 | 92.5 | 88.7 | 74.2 | 71.9 | 0.730 |
| ≥ 70% | 0.8472979 | 71.9 | 93.3 | 74.2 | 92.5 | 88.7 | 74.2 | 71.9 | 0.730 |
| ≥ 80% | 1.3862944 | 3.1 | 100.0 | 100.0 | 79.3 | 79.5 | 100.0 | 3.1 | 0.061 |
| ≥ 90% | 2.1972246 | 3.1 | 100.0 | 100.0 | 79.3 | 79.5 | 100.0 | 3.1 | 0.061 |
| ≥ 100% | Inf | 0.0 | 100.0 |  | 78.8 | 78.8 |  | 0.0 |  |
